# Association of exposure to urinary and blood heavy metals with visual disability among U.S. adults in NHANES 2013–2018

**DOI:** 10.3389/fpubh.2025.1583105

**Published:** 2025-05-09

**Authors:** Lingyu Dai, Qian Zhou, Yu Gao, Guannan Su, Qingyan Jiang, Lan Xia, Peizeng Yang

**Affiliations:** Ophthalmology Medical Center, The First Affiliated Hospital of Chongqing Medical University, Chongqing Key Laboratory for the Prevention and Treatment of Major Blinding Eye Diseases, Chongqing Branch (Municipality Division) of National Clinical Research Centre for Ocular Diseases, Chongqing, China

**Keywords:** heavy metals, visual disability, National Health and Nutrition Examination Survey, cadmium, lead, risk factors

## Abstract

**Background:**

Heavy metals exposure has been widely referred to as a risk factor for human health. However, studies on the potential impact of heavy metals on visual disability are limited. Herein, this study aims to investigate the associations of urinary and blood heavy metals with visual disability in adults.

**Methods:**

A total of 4,284 eligible participants in the 2013–2018 National Health and Nutrition Examination Survey (NHANES) were enrolled in our cross-sectional study. The urinary barium (Ba), cadmium (Cd), cesium (Cs), cobalt (Co), molybdenum (Mo), lead (Pb), antimony (Sb), thallium (Tl), tin (Sn), tungsten (Tu), and mercury (Hg) and blood Pb, Cd, and Hg were included for analysis. We used multivariate logistic regression, weighted quantile sum (WQS) regression, quantile-based gcomputation (qgcomp) regression, and Bayesian kernel machine regression (BKMR) to assess the mixed-metal effect on visual disability. The subgroup analysis was stratified by age.

**Results:**

In the single metal exposure model, the risk of visual disability increased by 39.2%, 22.6%, 25.6%, and 17.9% for each unit increase in urinary Cd, Pb, Sn, and Tu, respectively (all *p* < 0.05). Meanwhile, the risk of visual disability increased by 40.6% and 22.7% per unit increase in blood Ln-Pb and Ln-Cd, respectively (*p* = 0.034 and 0.018). In mixed metal effect analysis, WQS, qgcomp, and BKMR models consistently demonstrated a positive association between blood and urine metal co-exposure and visual disability. Furthermore, we found that Cd and Pb were the top-weighted metals responsible for the overall effect. However, these associations were not pronounced in the older adults.

**Conclusions:**

Our findings suggested that Cd, Pb, Sn, and Tu were identified as independent risk factors for visual disability. Furthermore, exposure to mixed metals could increase the risk of visual disability, to which Cd and Pb were the greatest contributors.

## 1 Introduction

Visual disability has been a long-standing and globally knotty public health issue, profoundly implicating individual, economic, and social life. Recently, the Vision Loss Expert Group (VLEG) of the Global Burden of Disease (GBD) Study reported approximately 43.3 million blindness and 295 million moderate to severe visual impairment population worldwide (1). The etiologies of irreversible vision loss are multifaceted and complex. It could not only be caused by ocular diseases such as glaucoma, cataracts, uveitis, diabetic retinopathy, and age-related macular degeneration (2, 3), but also by neurological disorders, systemic diseases, environmental intoxication, etc. (4, 5).

Heavy metals have infiltrated the environment and are ubiquitously present in our lives with the development of industrial technology and increasing serious pollution. Air, water, soil, food, and even dust are inevitably contaminated by heavy metals that are hazardous to human health. Several studies have indicated that exposure to heavy metals can lead to a variety of multi-system diseases, including ocular disorders, osteoarthritis, liver and renal dysfunction, cardiovascular diseases, rheumatoid arthritis, metabolic syndrome, tumors, neurodegenerative diseases, diabetes, etc. (6–13). Metals could enter the eye through oral route or direct contact with airborne ocular structures (e.g., tear film, cornea, conjunctiva) (14). Previous studies have revealed that long-term exposure to heavy metals can lead to various eye tissue damages, such as cell death in human lens epithelial cells and retinal pigment epithelial cells, retinal vascular dysfunction, and oxidative stress on retinal cells and neurons (15–18). Existing studies tended to focus on a single metallic effect on diseases that may amplify or disregard the co-exposure effects. However, a single metal cannot comprehensively illuminate the antagonistic or synergistic effects of multiple metals on human health due to the intricate multi-metal interactions in a realistic environment (19). To date, few epidemiological studies have evaluated the combined effects of multiple metal mixtures on visual disability.

Therefore, we conducted a cross-sectional study to investigate the single and mixed effects of 11 urinary metals and three blood metals on visual disability by using several regression models based on the National Health and Nutrition Examination Survey (NHANES) 2013-2018 in the U.S. population. This study provides novel epidemiological evidence on the associations between heavy metal exposures and visual disability. It may have a potential impact on mitigating preventable environmental risks and promoting ocular health.

## 2 Methods

### 2.1 Study design and enrollment

**Figure 1:**
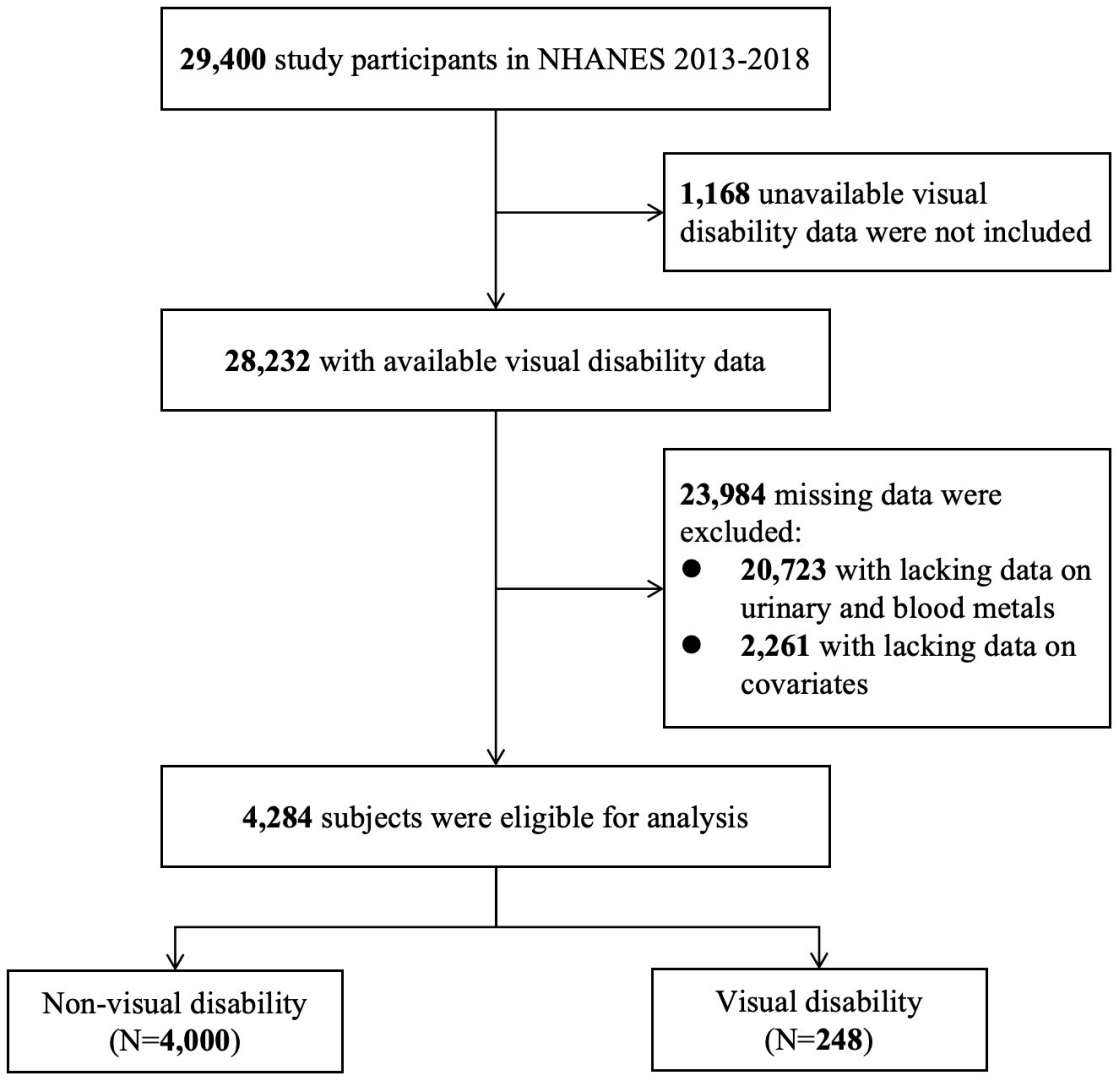
Flow diagram of enrollment and analysis populations.

NHANES is a national and cross-sectional survey aimed to assess the health and nutritional condition of the general population in the U.S. In this study, data were derived from 2013–2014, 2015–2016, and 2017–2018 NHANES. Figure 1 shows that 29,400 participants were screened. We excluded 1,168 subjects with unavailable visual disability data and 23,984 subjects with any missing data on urinary metals, blood metals, and covariates. A total of 4,284 eligible subjects were enrolled, including 4,000 with non-visual disability and 248 with visual disability. The programs in NHANES were all approved by the National Center for Health Statistics (NCHS) Ethic Review Board and all written informed consents were provided by participants before enrollment.

### 2.2 Assessment of vision disability

Visual disability is defined as a best-corrected visual acuity of < 3/60 or < 10° visual field around central fixation in the better eye (3). In this study, it was diagnosed by the disability questionnaire asked in the home by trained interviewers using the Computer-Assisted Personal Interview (CAPI) system. They were asked: “Are you blind?” or “Do you have serious difficulty seeing even when wearing glasses?” The participants who answered “Yes” were regarded as having a visual disability. However, those who answered “no” were considered to have a non-visual disability.

### 2.3 Metals measurement

The urinary barium (Ba), cadmium (Cd), cesium (Cs), cobalt (Co), molybdenum (Mo), lead (Pb), antimony (Sb), thallium (Tl), tin (Sn), tungsten (Tu), and mercury (Hg) and blood Pb, Cd, and Hg were measured by inductively coupled plasma mass spectrometry (ICP-MS). The ICP-MS method is a multi-element analytical technique capable of trace-level elemental analysis. The liquid samples were converted to an argon aerosol through ICP-MS and entered the mass spectrometer after atomization and ionization in a plasma field. The ions then passed through a focusing region, dynamic reaction cell, and the quadrupole mass filter. Finally, the ion detection signal was processed into digital data to quantify the concentration of the metallic elements (20). According to the NHANES standard, the metal concentration lower limit of detection was divided by the square root of two. Meanwhile, the concentrations of urinary metals were adjusted by urinary creatinine and calculated as μg/g creatinine.

### 2.4 Covariates

Covariates included gender, age at interview (20–59 years old, ≥60 years old), race/ethnicity (Mexican American, Other Hispanic, Non-Hispanic White, Non-Hispanic black, and Other Race/multiracial), educational level (below high school, high school or equivalent, and above high school), marital status (married/living with partner, widowed/divorced/separated, and never married), body mass index (BMI) (< 25, 25–29.9, ≥30 kg/m^2^), family income to poverty (PIR), serum cotinine, and NHANES cycles. Serum cotinine concentrations identifying exposure to tobacco smoke were categorized into active/secondhand smokers and nonsmokers with a cutoff of 0.11 ng/mL (21).

### 2.5 Statistical analysis

All statistical analyses were performed by STATA (version 17.0) and R (version 4.3.1). Chi-square and Mann-Whitney U tests were used to assess the basic demographic characteristics of participants. The concentrations of heavy metals (continuous variables) were Ln-transformed to obtain similarly normal distributions, and divided into four quartiles (Q1, Q2, Q3, and Q4) as categorical variables. Pearson correlation analysis was used to evaluate the correlation of Ln-transformed heavy metal concentrations in urine and blood, in which the coefficients above 0.3 were considered to be correlating. We applied multivariable logistic regression to calculate odds ratios (ORs) and 95% confidence intervals (CIs) to estimate the effects of different metals on visual disability.

The Weight quantile sum (WQS) regression was carried out to explore the total mixture effect of multi-metals on visual disability and reveal the predominant exposure. R package (gWQS) can calculate the WQS index comprised of the weighted sums of specific metal concentrations. The WQS index, with a range of 0 to 1, reflects the contribution of each metal to the co-exposure effect in urine or blood. We randomly divided data into training and validation sets, respectively accounting for 40% and 60%. The training dataset underwent 1,000 iterations of bootstrap to estimate the weight of each metal, while the validation dataset was utilized to assess the significance of the WQS index.

The BKMR model was used to address the non-linearity and non-additive dose-response correlations among multi-variables exposure. Posterior inclusion probability (PTP) was used to assess the contribution of each component to the outcomes, with a value >0.5 being deemed significant. The overall effect plot illustrates the combined effect of all metals at a specific percentile as compared to when all of them are at their 50th percentile. The single exposure-response plots demonstrate the impact of a single metal on the outcome at different quartiles when the remaining elements are fixed at the 25th, 50th, and 75th percentiles. Binary exposure-response curves reflect the relationship of two exposures. It refers to the potential interaction between the 25th, 50th, and 75th percentiles of one metal and another when the remaining variables are fixed at the median. The iteration in the BKMR model was set at 25,000 and run by “bkmr” package in R software.

Sensitivity analysis and subgroup analysis were performed to robust the estimations of models. The quantile-based g computation (qgcomp) model was used to overcome the restriction of the WQS model that estimates outcome-related exposures in the same direction. Qgcomp regression allowing heterogeneous directions was used to calculate the positive and negative weights of each index of the mixtures by R package “qgcomp”. Furthermore, subgroup analysis was conducted stratified by age to adjust model bias. All models were corrected by gender, age, race/ethnicity, educational level, marital status, BMI, PIR, serum cotinine, and NHANES cycles.

## 3 Result

### 3.1 Population characteristics, heavy metals distributions, and correlations

Among the 4,284 participants in the 2013–2018 NHANES, 284 (6.63%) had a visual disability. Table 1 summarizes the demographic characteristics of participants among U.S. adults. There were statistically significant differences between non-visual disability and visual disability subjects in age, race/ethnicity, education level, marital status, family PIR, and serum cotinine (*p* < 0.05). However, the two groups had a comparable gender and BMI.

**Table 1:** Basic characteristics of subjects by visual disability among U.S. adults in the NHANES 2013–2018.

	**Subjects**			
**Characteristics**	**Overall (*****N*** = **4,284)**	**Non-visual disability (*****N*** = **4,000)**	**Visual disability (*****N*** = **284)**	***P*** **value**
**Gender**, ***n*** **(%)**				0.719
Male	2,077 (48.89)	1,953 (48.83)	124 (50)	
Female	2,171 (51.11)	2,047 (51.18)	124 (50)	
**Age, year**, ***n*** **(%)**				< 0.001
20–59	2,835 (66.74)	2,733 (68.33)	102 (41.13)	
≥60	1,413 (33.26)	1,267 (31.67)	146 (58.87)	
**Race/ethnicity**, ***n*** **(%)**				0.005
Mexican American	609 (14.34)	558 (13.95)	51 (20.56)	
Other Hispanic	435 (10.24)	406 (10.15)	29 (11.69)	
Non-Hispanic White	1,683 (39.62)	1,585 (39.62)	98 (39.52)	
Non-Hispanic Black	845 (19.89)	798 (19.95)	47 (18.95)	
Other race/multiracial	676 (15.91)	653 (16.32)	23 (9.27)	
**Educational level**, ***n*** **(%)**				< 0.001
Below high school	842 (19.82)	744 (18.6)	98 (39.52)	
High school or equivalent	998 (23.49)	929 (23.23)	69 (27.82)	
Above high school	2,408 (56.69)	2,327 (58.17)	81 (32.66)	
**Marital status**, ***n*** **(%)**				< 0.001
Married/living with partner	2,577 (60.66)	2,449 (61.22)	128 (51.61)	
Widowed/divorced/separated	904 (21.28)	814 (20.35)	90 (36.29)	
Never married	767 (18.06)	737 (18.43)	30 (12.1)	
**BMI, kg/m**^2^, ***n*** **(%)**				0.116
< 25	1,158 (27.26)	1,101 (27.52)	57 (22.98)	
25–29.9	1,373 (32.32)	1,297 (32.42)	76 (30.65)	
≥30	1,717 (40.42)	1,602 (40.05)	115 (46.37)	
**Serum cotinine, ng/mL**, ***n*** **(%)**				0.001
≤ 0.011	1,385 (32.6)	1,327 (33.17)	58 (23.39)	
>0.011	2,863 (67.4)	2,673 (66.83)	190 (76.61)	
**Family PIR**, ***n*** **(%)**				< 0.001
≤ 1.30	1,422 (33.47)	1,296 (32.4)	126 (50.81)	
1.31–3.50	1,720 (40.49)	1,630 (40.75)	90 (36.29)	
>3.50	1,106 (26.04)	1,074 (26.85)	32 (12.9)	

BMI, body mass index; NHANES, National Health and Nutrition Examination Survey; PIR, Poverty Income Ratio.

### 3.2 Distribution and correlations of urinary and blood metals

The distributions of urinary and blood metal concentrations are presented in Supplementary Table S1. The detection rates of urinary and blood metal concentrations were above 80.0% except for Hg in urine. Pearson's correlation matrix displayed the correlation between these Ln-transformed metals (Supplementary Figure S1). In urine, there was a positive correlation between Ba and Co (*r* = 0.46), Cd and Pb (*r* = 0.42), Co and Cs (*r* = 0.39), Co and Tl (*r* = 0.33), Cs and Pb (*r* = 0.34), Cs and Tl (*r* = 0.63), Mo and Tu (0.40). A mild correlation was found between blood Pb and blood Cd (*r* = 0.33). The correlations of others were comparatively weak.

### 3.4 The association of single metals with visual disability disclosed by multivariate logistic regression

Table 2 shows the multivariate logistic regression outcomes adjusted by all covariates. For each unit increase in urinary Ln-Cd, Ln-Pb, Ln-Sn, and Ln-Tu, there was a 39.2%, 22.6%, 25.6%, and 17.9% increase in the risk of visual disability, respectively (all *p* < 0.05). For the highest exposure level (Q4), the concentrations of urinary Cd, Co, Pb, and Sn significantly increased visual disability risk as compared to the lowest level (Q1) (Cd: OR = 2.032, 95%CI: 1.280–3.228; Co: OR = 1.558, 95%CI: 1.028–2.362; Pb: OR = 1.708, 95%CI: 1.109–2.632; Sn: OR = 3.047, 95%CI: 1.446–6.418). Urinary Cd and Sn of the Q3 level were also found to significantly increase the risk of visual disability as compared to that of the Q1 level (Cd: OR = 1.638, 95%CI: 1.038–2.584; Sn: OR = 2.447, 95%CI: 1.164–5.143). The risk of visual disability rose by 40.6% and 22.7% per unit increase in blood Ln–Pb and Ln–Cd, respectively (*p* = 0.034 and 0.018). The blood Pb of the Q4 level had a greater impact on visual disability compared to the Q1 level (OR = 1.580, 95% CI:1.014–2.462). Moreover, blood Cd in the Q3 and Q4 levels both significantly increased visual disability risk compared to that in the Q1 level (Q3: OR = 1.707,95%CI:1.136–2.565; Q4: OR = 1.568, 95%CI:1.026–2.398).

**Table 2:** Associations of single urinary and blood metals with visual disability in the NHANES 2013–2018.

	**Continuous**		**Q1**	**Q2**		**Q3**		**Q4**	
	**OR (95%CI)**	***p*** **value**		**OR (95%CI)**	***p*** **value**	**OR (95%CI)**	***p*** **value**	**OR (95%CI)**	***p*** **value**
**Urine (**μ**g/g creatinine)**									
**Ba**									
Total	0.880 (0.769–1.007)	0.062	Reference	0.917 (0.643–1.309)	0.633	0.657 (0.444–0.971)	0.035	0.819 (0.564–1.188)	0.293
20–59	0.885 (0.712–1.101)	0.274	Reference	0.521 (0.282–0.960)	0.037	0.644 (0.369–1.125)	0.122	0.757 (0.436–1.312)	0.320
≥60	0.870 (0.733–1.032)	0.110	Reference	1.268 (0.808–1.992)	0.302	0.628 (0.362–1.089)	0.098	0.827 (0.498–1.375)	0.464
**Cd**									
Total	1.392 (1.172–1.654)	< 0.001	Reference	1.264 (0.790–2.022)	0.328	1.638 (1.038–2.584)	0.034	2.032 (1.280–3.228)	0.003
20–59	1.597 (1.242–2.053)	< 0.001	Reference	1.351 (0.727–2.513)	0.341	1.775 (0.946–3.331)	0.074	2.723 (1.443–5.138)	0.002
≥60	1.186 (0.932–1.511)	0.166	Reference	0.916 (0.437–1.92)	0.816	1.104 (0.553–2.204)	0.778	1.267 (0.638–2.519)	0.499
**Co**									
Total	1.071 (0.875–1.311)	0.508	Reference	1.680 (1.137–2.482)	0.009	1.037 (0.679–1.584)	0.867	1.558 (1.028–2.362)	0.036
20–59	0.947 (0.661–1.357)	0.768	Reference	1.426 (0.797–2.551)	0.232	0.877 (0.456–1.689)	0.695	1.354 (0.71–2.582)	0.358
≥60	1.111 (0.871–1.417)	0.396	Reference	1.872 (1.097–3.194)	0.021	1.168 (0.665–2.049)	0.589	1.676 (0.959–2.928)	0.070
**Cs**									
Total	0.814 (0.610–1.087)	0.163	Reference	0.890 (0.612–1.296)	0.545	1.016 (0.696–1.483)	0.934	0.758 (0.497–1.156)	0.198
20–59	0.722 (0.464–1.125)	0.150	Reference	0.901 (0.523–1.550)	0.706	0.84 (0.464–1.522)	0.565	0.749 (0.393–1.425)	0.378
≥60	0.870 (0.593–1.276)	0.476	Reference	0.861 (0.511–1.453)	0.576	1.098 (0.665–1.812)	0.716	0.752 (0.428–1.321)	0.322
**Mo**									
Total	1.057 (0.861–1.297)	0.596	Reference	0.986 (0.669–1.454)	0.945	1.102 (0.752–1.616)	0.618	0.999 (0.676–1.476)	0.995
20–59	1.109 (0.803–1.530)	0.530	Reference	0.768 (0.432–1.364)	0.368	0.777 (0.425–1.421)	0.413	1.208 (0.692–2.108)	0.506
≥60	1.039 (0.797–1.355)	0.776	Reference	1.259 (0.738–2.148)	0.398	1.417 (0.848–2.368)	0.183	0.929 (0.538–1.604)	0.792
**Pb**									
Total	1.226 (1.022–1.472)	0.028	Reference	1.206 (0.767–1.897)	0.418	1.415 (0.914–2.19)	0.119	1.708 (1.109–2.632)	0.015
20–59	1.405 (1.081–1.825)	0.011	Reference	1.546 (0.837–2.856)	0.164	1.48 (0.78–2.811)	0.230	2.391 (1.295–4.416)	0.005
≥60	1.049 (0.814–1.353)	0.712	Reference	0.756 (0.381–1.5)	0.424	0.986 (0.53–1.834)	0.964	1.062 (0.576–1.96)	0.846
**Sb**									
Total	1.097 (0.908–1.326)	0.336	Reference	1.228 (0.814–1.853)	0.328	1.539 (1.037–2.283)	0.032	1.429 (0.957–2.132)	0.081
20–59	1.119 (0.855–1.465)	0.412	Reference	1.681 (0.873–3.239)	0.120	1.77 (0.926–3.385)	0.084	1.892 (1.003–3.569)	0.049
≥60	1.094 (0.837–1.429)	0.510	Reference	0.994 (0.581–1.701)	0.983	1.45 (0.877–2.397)	0.148	1.200 (0.708–2.035)	0.499
**Sn**									
Total	1.256 (1.110–1.421)	< 0.001	Reference	1.287 (0.791–2.094)	0.309	2.447 (1.164–5.143)	0.018	3.047 (1.446–6.418)	0.003
20–59	1.274 (1.055–1.539)	0.012	Reference	0.991 (0.41–2.395)	0.984	1.85 (0.581–5.886)	0.297	3.167 (0.994–10.091)	0.051
≥60	1.234 (1.045–1.457)	0.013	Reference	1.412 (0.768–2.596)	0.266	2.464 (0.862–7.049)	0.093	2.474 (0.874–7.005)	0.088
**Tl**									
Total	0.829 (0.657–1.047)	0.116	Reference	0.732 (0.510–1.049)	0.089	0.713 (0.491–1.034)	0.074	0.819 (0.555–1.208)	0.314
20–59	0.798 (0.55–1.157)	0.234	Reference	0.841 (0.483–1.464)	0.539	0.791 (0.439–1.425)	0.436	0.875 (0.482–1.589)	0.661
≥60	0.873 (0.647–1.179)	0.376	Reference	0.684 (0.425–1.101)	0.118	0.684 (0.422–1.109)	0.123	0.824 (0.492–1.382)	0.463
**Tu**									
Total	1.179 (1.015–1.368)	0.031	Reference	1.096 (0.746–1.610)	0.641	1.026 (0.69–1.524)	0.901	1.432 (0.985–2.081)	0.060
20–59	1.138 (0.903–1.434)	0.273	Reference	0.696 (0.374–1.295)	0.253	0.952 (0.532–1.702)	0.867	1.281 (0.741–2.214)	0.375
≥60	1.228 (1.010–1.493)	0.039	Reference	1.517 (0.918–2.508)	0.104	1.126 (0.655–1.936)	0.666	1.635 (0.98–2.727)	0.060
**Hg**									
Total	0.996 (0.875–1.134)	0.956	Reference	1.015 (0.622–1.655)	0.953	1.363 (0.708–2.622)	0.354	1.17 (0.595–2.301)	0.648
20–59	0.993 (0.816–1.209)	0.944	Reference	1.140 (0.559–2.324)	0.719	2.455 (0.88–6.847)	0.086	1.621 (0.55–4.776)	0.381
≥60	0.993 (0.833–1.183)	0.936	Reference	0.899 (0.460–1.760)	0.757	0.835 (0.35–1.992)	0.685	0.913 (0.376–2.215)	0.840
**Blood (**μ**g/L)**									
**Pb**									
Total	1.406 (1.143–1.731)	< 0.001	Reference	1.086 (0.688–1.713)	0.723	1.295 (0.83–2.019)	0.255	1.580 (1.014–2.462)	0.043
20–59	1.617 (1.209–2.162)	< 0.001	Reference	1.117 (0.607–2.055)	0.723	1.386 (0.749–2.564)	0.298	2.231 (1.225–4.065)	0.009
≥60	1.176 (0.869–1.591)	0.295	Reference	0.79 (0.382–1.633)	0.525	0.886 (0.447–1.754)	0.728	0.939 (0.476–1.856)	0.857
**Cd**									
Total	1.227 (1.036–1.454)	0.018	Reference	1.332 (0.851–2.084)	0.209	1.707 (1.136–2.565)	0.010	1.568 (1.026–2.398)	0.038
20–59	1.225 (0.971–1.544)	0.087	Reference	1.353 (0.696–2.632)	0.373	1.623 (0.866–3.041)	0.131	1.544 (0.845–2.822)	0.158
≥60	1.141 (0.885–1.471)	0.310	Reference	1.188 (0.642–2.201)	0.583	1.6 (0.926–2.765)	0.092	1.363 (0.745–2.494)	0.315
**Hg**									
Total	0.922 (0.796–1.067)	0.276	Reference	1.205 (0.843–1.724)	0.307	1.105 (0.767–1.594)	0.592	0.887 (0.586–1.344)	0.573
20–59	1.045 (0.841–1.298)	0.692	Reference	1.812 (1.035–3.174)	0.038	1.591 (0.889–2.845)	0.118	1.463 (0.771–2.778)	0.245
≥60	0.832 (0.681–1.017)	0.073	Reference	0.899 (0.560–1.444)	0.660	0.871 (0.539–1.406)	0.571	0.610 (0.351–1.061)	0.080

Continuous, Ln-transformed concentration of metal; CI, confidence interval; OR, odds ratio; Q, quartile. Models were adjusted for age, gender, race/ethnicity, education levels, marital status, body mass index, serum cotinine, and family poverty income ratio.

In the 20–59 age group, an increase in urinary Ln–Cd, Ln–Pb, Ln–Sn, and blood Ln–Pb per unit enhanced the risk of visual disability by 59.7%, 40.5%, 27.4%, and 61.7%, respectively (all *p* < 0.05). Compared to the Q1 level, urinary Cd, Pb, and blood Pb of Q4 level significantly improved visual disability risk (Urinary Cd: OR = 2.723,95% CI: 1.443–5.138; Urinary Pb: OR = 2.391, 95%CI: 1.295–4.416; Blood Pb: OR = 2.231, 95%CI:1.225–4.065). Among individuals aged 60 and above, there was a notable positive association between urinary Sn and Tu with visual disability (Sn: OR = 1.234, 95%CI: 1.045–1.457; Tu: OR = 1.228, 95%CI: 1.01–1.493). No significant correlations between other metals and visual disability were found.

### 3.5 The mixed effect of multiple metals on visual disability disclosed by WQS and qgcomp regression model

**Figure 2:**
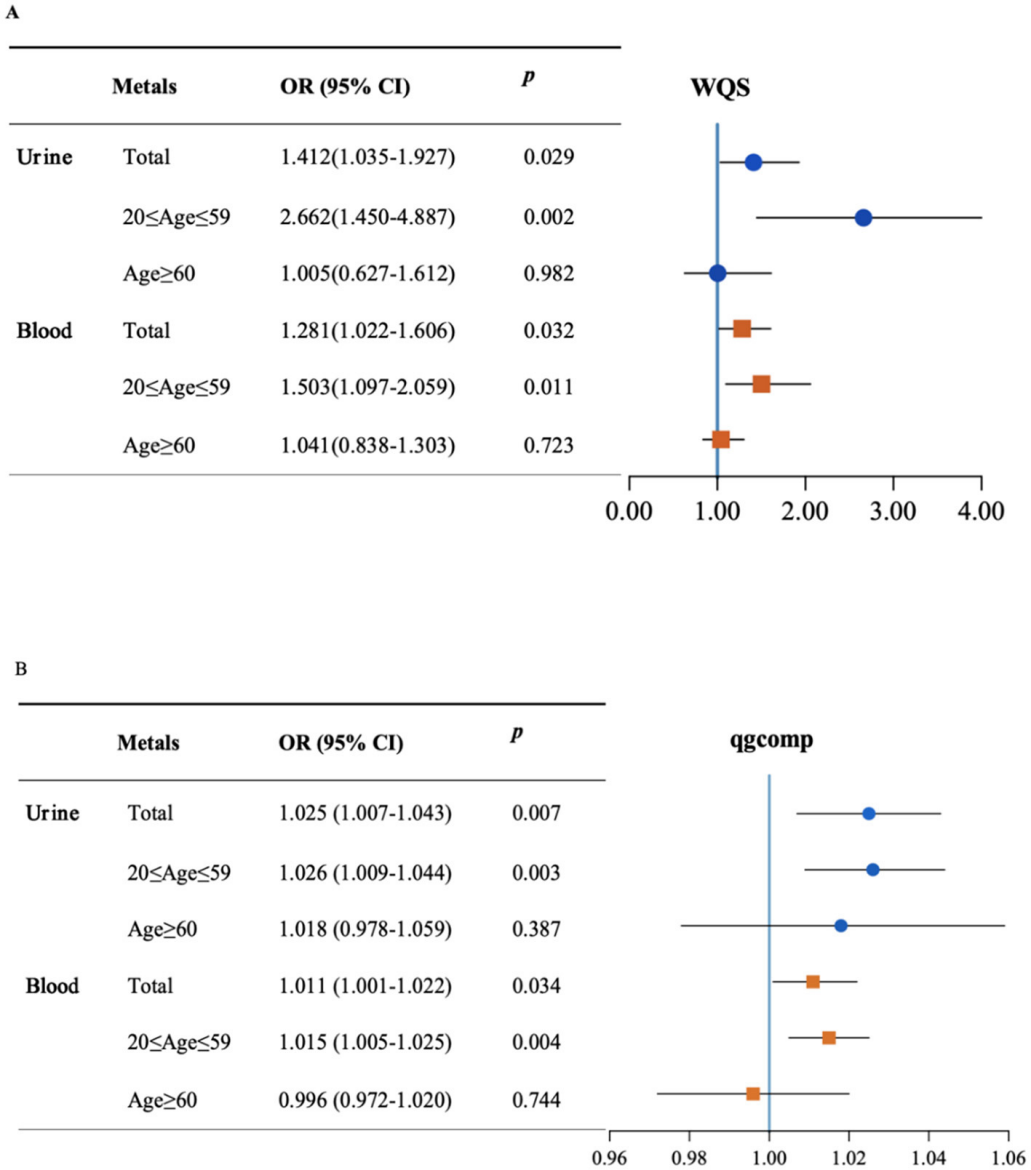
The mixed effect of metals on visual disability and stratified by age. Weight quantile sum (WQS) model **(A)** and quantile-based g computation (qgcomp) model **(B)** were adjusted for age, gender, race/ethnicity, education levels, poverty income ratio, marital status, body mass index, serum cotinine, and NHANES cycles.

The WQS model corrected by all covariates showed a substantial association of per–unit increase in urinary and blood metal co–exposure with the risk of visual disability for the total population (Urine: OR = 1.412, 95%CI: 1.035–1.927; Blood: OR = 1.281, 95%CI: 1.022–1.606) (Figure 2A). In the subgroup analysis, the positive effects of urinary and blood metal co–exposure on visual disability were more pronounced in the 20–59 age group (Urine: OR = 2.662, 95%CI: 1.450–4.887; Blood: OR = 1.503, 95%CI: 1.097–2.059), but not statistically significant in the ≥60 age group (Urine: OR = 1.005, 95%CI: 0.627–1.612; Blood: OR = 1.041, 95%CI: 0.838–1.303). Supplementary Figure S2 displays the WQS index of each metal. Cd accounted for the heaviest weight in the urine and blood metal mixtures for all groups, except for blood Pb, which was the top–weighted metal in the blood metal mixture for the 20–59 age group.

In the sensitivity analysis, Figure 2B shows the qgcomp model outcome adjusted by all confounders. The effect of urinary metal co–exposure was still significantly associated with the risk of visual disability in the total population (OR = 1.025, 95%CI: 1.007–1.043) as well as in the 20–59 age group (OR = 1.026, 95%CI: 1.009–1.044). However, the overall effect was not significant in the ≥60 age group (OR = 1.018, 95%CI: 0.978–1.059). The correlations between blood metals and visual disability risk were also significant in the total population and the 20–59 age group (OR = 1.011, 95%CI: 1.001–1.022; OR = 1.015, 95%CI: 1.005–1.025, respectively), whereas not statistically significant in individuals aged 60 and above (OR = 0.996, 95%CI: 0.972–1.02). The positive and negative directions of the estimated exposure weights are presented in Supplementary Figure S3, in which Cd and Pb were the main positive contributors to mixed exposure in urine and blood. However, Ba was the negative top–weighted metal in urine metal exposure and Hg was the negative highest–weighted metal in blood mixtures.

### 3.6 The associations of mixed metals exposure and visual disability disclosed by BKMR regression model

**Figure 3:**
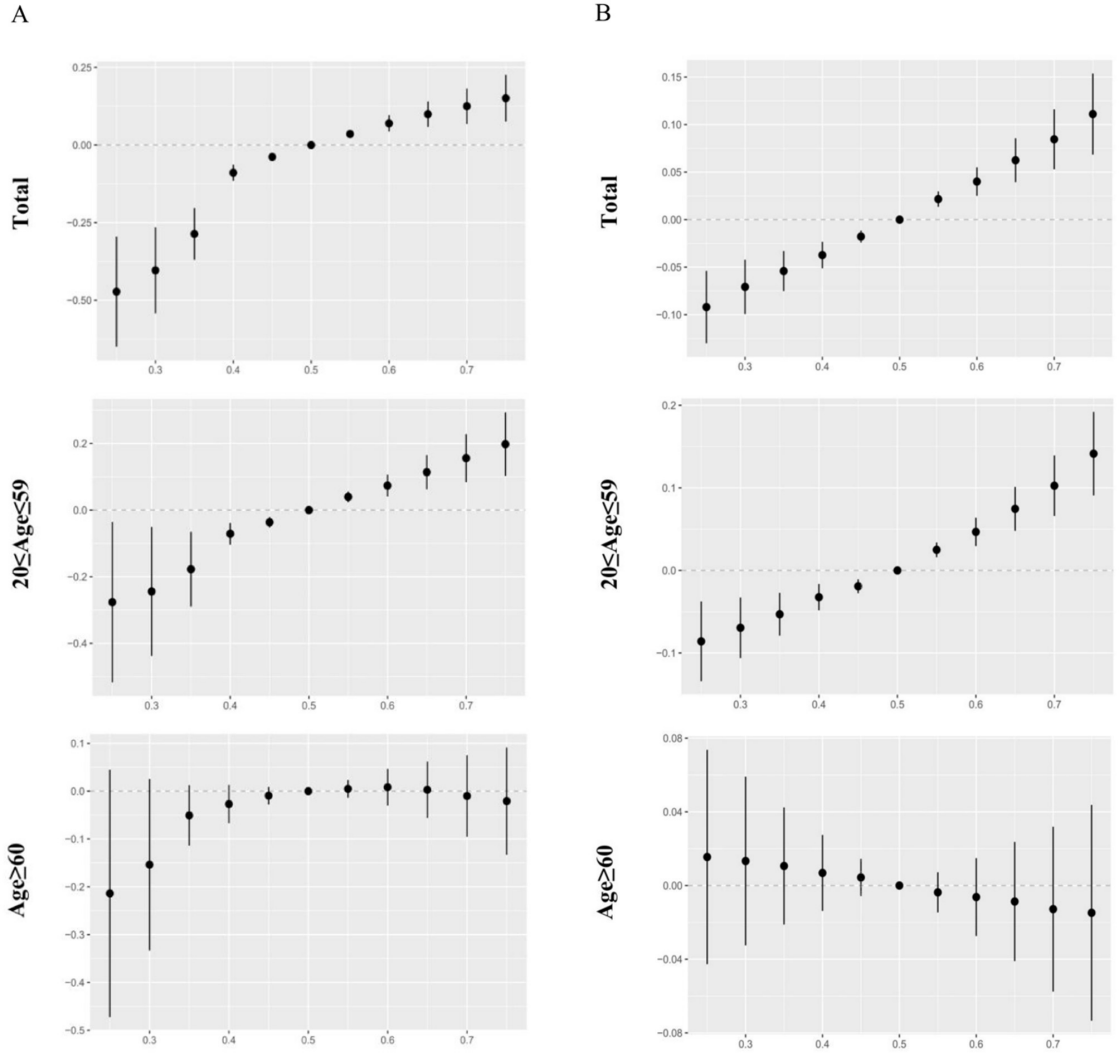
The overall effects of urinary and blood metal mixtures on visual disability were estimated by Bayesian Kernel Machine Regression (BKMR) models when all the metals at particular percentiles were compared to those at their 50th percentile. Models were adjusted for age, gender, race/ethnicity, education levels, poverty income ratio, marital status, body mass index, serum cotinine, and NHANES cycles. **(A)** Mixed effects of urinary metals. **(B)** Mixed effects of blood metals.

The effect of urinary and blood metals on visual disability estimated by BKMR regression is displayed in Figure 3. When the concentrations of metals were at or above the 55th percentile, the urinary and blood co-exposure effects both significantly increased the risk of visual disability as compared to the 50th percentile. Significantly similar upward trend effects were observed in the 20–59 age group, but not found in the ≥60 age group. Supplementary Table S2 shows that the Cd, Cs, Pb, Sn, and Tu in urine and Pb and Cd in blood had a PIP value greater than 0.5 in the total population. We also found that the PTP values of urinary Cd, blood Pb, and blood Cd were above 0.5 in the 20-59 age group, whereas no PIP value above 0.5 was found in the ≥60 age group. Furthermore, when other metals respectively fixed at the 25th, 50th, and 75th percentile, urinary Hg, Sn, Cd and blood Cd, Pb showed notable effects on visual disability in the total population.

**Figure 4:**
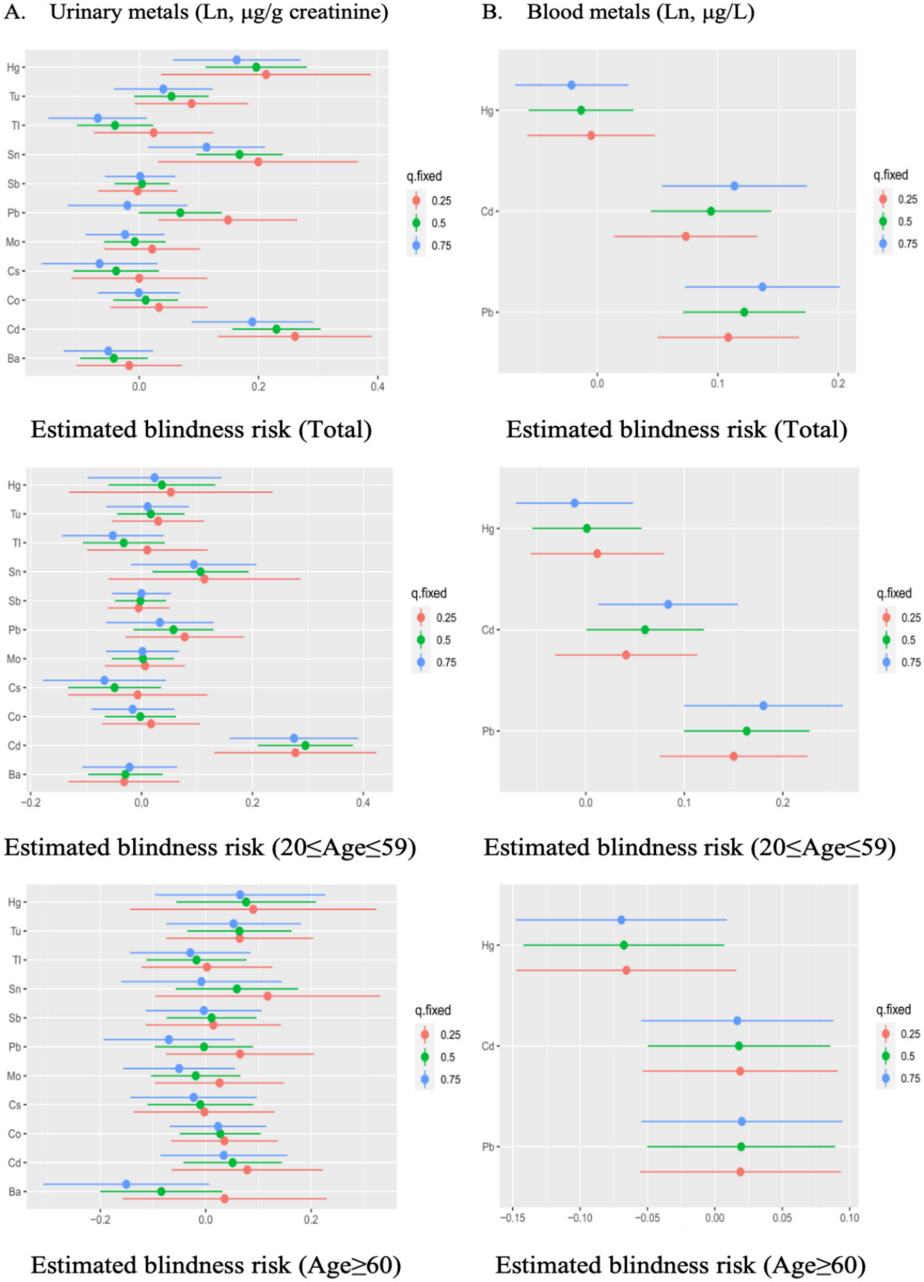
The estimated effect of single urinary **(A)** and blood **(B)** metals on visual disability in BKMR regression models, when other all metals were held at their corresponding 25th (red), 50th (green), or 75th (blue) percentile, respectively. Models were adjusted for gender, age, race/ethnicity, education levels, poverty income ratio, marital status, body mass index, serum cotinine, and NHANES cycles.

In the 20–59 age participants, urinary Cd and blood Pb were found to be positively correlated with visual disability when other metals were fixed at the 25th, 50th, and 75th percentile. Additionally, blood Cd significantly affected visual disability whenever other metals were fixed at the 50th and 75th percentile. However, no significant exposure was found in the older adults group (Figure 4). When the concentrations of other metals were fixed at the median, the single concentration-response plots (Supplementary Figure S4) demonstrated a gradual increase in the advancement of visual disability caused by urinary Cd and blood Pb. Supplementary Figure S5 indicates that there may be underlying interactions between several metals in visual disability, such as urinary Cd and Co, Pb, Sn, urinary Hg and Tl, Tu, blood Pb and Cd, Hg.

## 4 Discussion

To our knowledge, it is the first study to investigate the possible correlation of multiple urinary and blood metals on visual disability in the U.S. population. For the single metal exposure logistic regression models, urinary Cd, Pb, Sn, and Tu as well as blood Pb and Cd were found to significantly increase the risk of visual disability. For the mixed metal exposure analysis, WQS, qgcomp, and BKMR regression models consistently showed a positive correlation between the mixed effect of urinary and blood metals and visual disability. Cd and Pb were identified as the highest-weighted metals in the mixed effect of metals. Moreover, the combined metal effects were only found in the young and middle age group, but not in the older adults group.

Previous studies have revealed that the accumulation levels of toxic heavy metals in the RPE, choroid, iris, and ciliary body were higher than those in the blood, suggesting that these eye tissues have a strong affinity for metal ions (22, 23). Cd and Pb have been listed as some of the 10 most hazardous heavy metals (24). Cd has been widely distributed in atmosphere, drinking water, smoking, food, soil, and industrial products. The biological half-life of Cd in human organs is up to 10–30 years with a high residence and low clearance (25). In this study, we found that increased urinary and blood Cd were both associated with the risk of visual disability, and this result is consistent with previous studies, in which exposure to Cd could lead to blind-causing diseases such as glaucoma, cataracts, optic neuritis, and age-related macular degeneration, eventually seriously threatening visual acuity (26–28). Pieces of evidence further supported our results, Cd has the potential to damage human retinal pigment epithelium (RPE) cells, photoreceptors, and optic nerve by generating reactive oxygen species, inducing oxidative stress and endoplasmic reticulum stress, producing inflammatory cytokines, and damaging vascular system (29–32). It is worthwhile to point out that these mechanism studies were based on exposure models with high Cd concentrations (e.g., cellular experiments: 10–100 μM; animal experiments: 1–5 mg/kg), which far exceed the actual exposure levels of the visual disability population in NHANES [median blood Cd: 0.41 (0.23-0.71) μg/L, median urinary Cd: 0.31 (0.15–0.58) μg/g creatinine]. Although the exposure doses were different, the pathogenic pathways identified in these studies have been shown to have a cumulative effect at low doses of long-term Cd exposure (33), which provided biological plausibility for our observational associations. Furthermore, we also found that Pb was a risk factor for visual disability, which has widely existed in batteries, gasoline, paints, and various industrial processes. Individuals with long-term exposure to Pb had lower macular, choroidal, and retinal nerve fiber thicknesses as compared to healthy controls (34). Pb not only tightly binds with sulfhydryl and decreases glutathione by inhibiting the activity of antioxidant enzymes (35), but also competitively inhibits the calcium channels into neurons and retina and impairs nervous transmission (36). Numerous shreds of evidence also proved that accumulation of Pb on the optic nerve and retina could cause optic neuropathy and retinopathy, eventually leading to vision loss (37–39). In addition to Cd and Pb, urinary Sn and Tu were found to increase the risk of visual disability in our study. Sn and Tu, as industrial and environmental toxicants, were known to cause pulmonary inflammation, cardiometabolic dysfunction, neurological disorders, and autoimmune disease (40, 41). Tu can often augment the effects of other co-exposures or co-stressors, potentially resulting in greater toxicity or more severe diseases (42). However, experimental research on the links of these metals to vision dysfunction is less conducted.

Humans are concurrently exposed to multiple metals and diseases are caused by synergistic and antagonistic interactions of metals. Previous studies on the association between mixed metal effects and vision are comparatively small. An animal experiment discovered that Cd and Pb exposure could significantly damage vision, but their combined exposure caused an opposite effect (43). Our findings extend the knowledge about the impact of co-exposure metals on visual disability. The WQS model estimating in the same direction found that the mixed metal effect significantly increased the risk of visual disability. The outcomes of qgcomp model without restriction on the direction were comparable to the results of WQS regression, which further ensured the reliability of our outcomes. The nonlinear and non-additive relationship between metals disclosed by the BKMR model further implied that the mixed effect had a dose-response association with the risk of visual disability. These three models all revealed that Cd and Pb were primarily responsible for the overall co-exposure effect. Interestingly, in the subgroup analysis, a significant association between the overall effect of urinary or blood metals and visual disability was only found in the 20–59 age group, but not in the older adults group. A potential explanation is that young and middle-aged people are more exposed to various toxic metals, such as smoking, burning materials, batteries, mining, and industrial activity.

The toxic effect of metals on visual disability has remained poorly understood, but oxidative stress, DNA damage, and lipid peroxidation are generally regarded as important factors in driving vision loss (44–46). Furthermore, recent studies have suggested that metals could induce the generation of pro-inflammatory cytokines, including TNF-α, IL-1β, IL-6, IL-10, and IL-12 (47, 48). These mechanisms of metals may be involved in the processes of blind-causing diseases (49–52). Nevertheless, the elucidation of underlying mechanisms of metal interactions on the pathogenesis of vision loss deserves further research.

This study conducted several novel statistical models to assess the possible associations between heavy metals and visual disability, which robustly made our conclusions more reliable. Moreover, the study was carried out in a relatively large population and all data were subject to strict quality control measures of NHANES. However, there were also some inevitable limitations. Firstly, due to the design of a cross-sectional study, the associations between heavy metals and visual disability found in this study were a potential epidemiological link. It could not reflect a temporal causal relationship between metal exposure and the risk of vision loss. These findings should be further confirmed in depth by longitudinal studies and laboratory studies. Secondly, although some covariates were corrected, there are still uncontrollable and residual confounders, and the results may also be affected. In addition, due to the lack of related genetic data in the NHANES database, genetic factors (e.g. a family history of visual disability and genetic factors leading to blinding ocular diseases) were not included in our analysis. Therefore, future studies could focus on integrating genetics data and environmental exposomics to assess the effect of genetic predisposition on the associations between heavy metals and visual disability. Thirdly, each urinary and blood sample was only measured one time, which may cause measurement errors due to individual differences in metabolite excretion and half-life of each metal.

## 5 Conclusion

Our findings demonstrated that Cd, Pb, Sn, and Tu in urine, as well as Pb and Cd in blood, were significantly associated with an increased risk of visual disability. Furthermore, the mixed metal effect analysis indicated a positive association between metal mixture exposure and visual disability, in which Cd and Pb were the dominant positive contributors. However, due to the limitations of this study, future prospective cohort studies with longitudinal heavy metal monitoring and clinical validation are needed to further confirm these associations.

## Data Availability

The original contributions presented in the study are included in the article/Supplementary material, further inquiries can be directed to the corresponding author.
