# Supplementary material for: Association of exposure to urinary and blood heavy metals with visual disability among U.S. adults in NHANES 2013–2018
Source: Front Public Health. 2025 May 9;13:1583105. doi: 10.3389/fpubh.2025.1583105 (PMC12098593; doi:10.3389/fpubh.2025.1583105)
Supplement: Supplementary file 1 [file Data_Sheet_1.docx]

***Supplementary material***


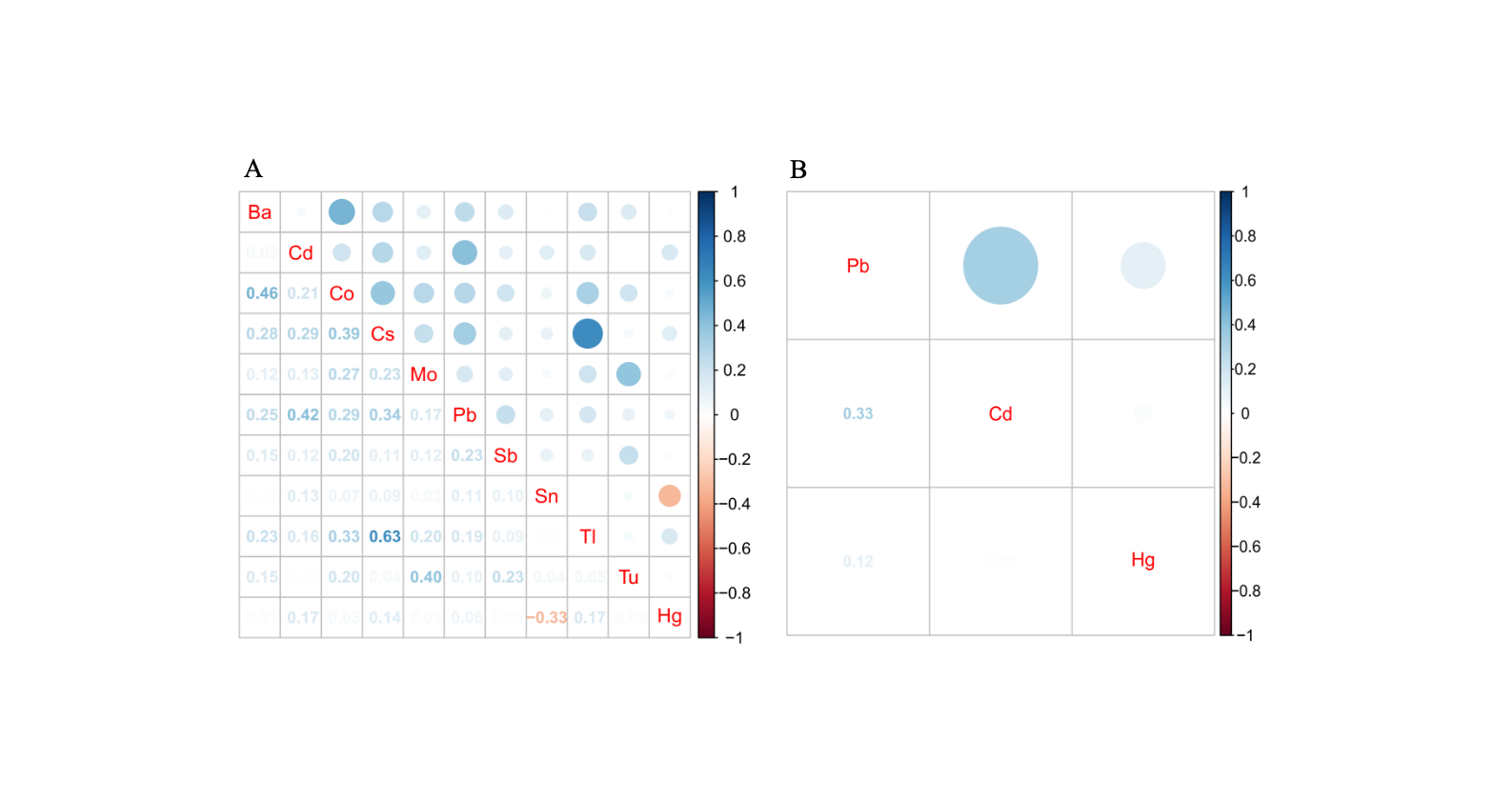
Figure S1. Pearson’s correlation matrix among Ln-transformed urinary (A) and blood (B) metals in the study population.


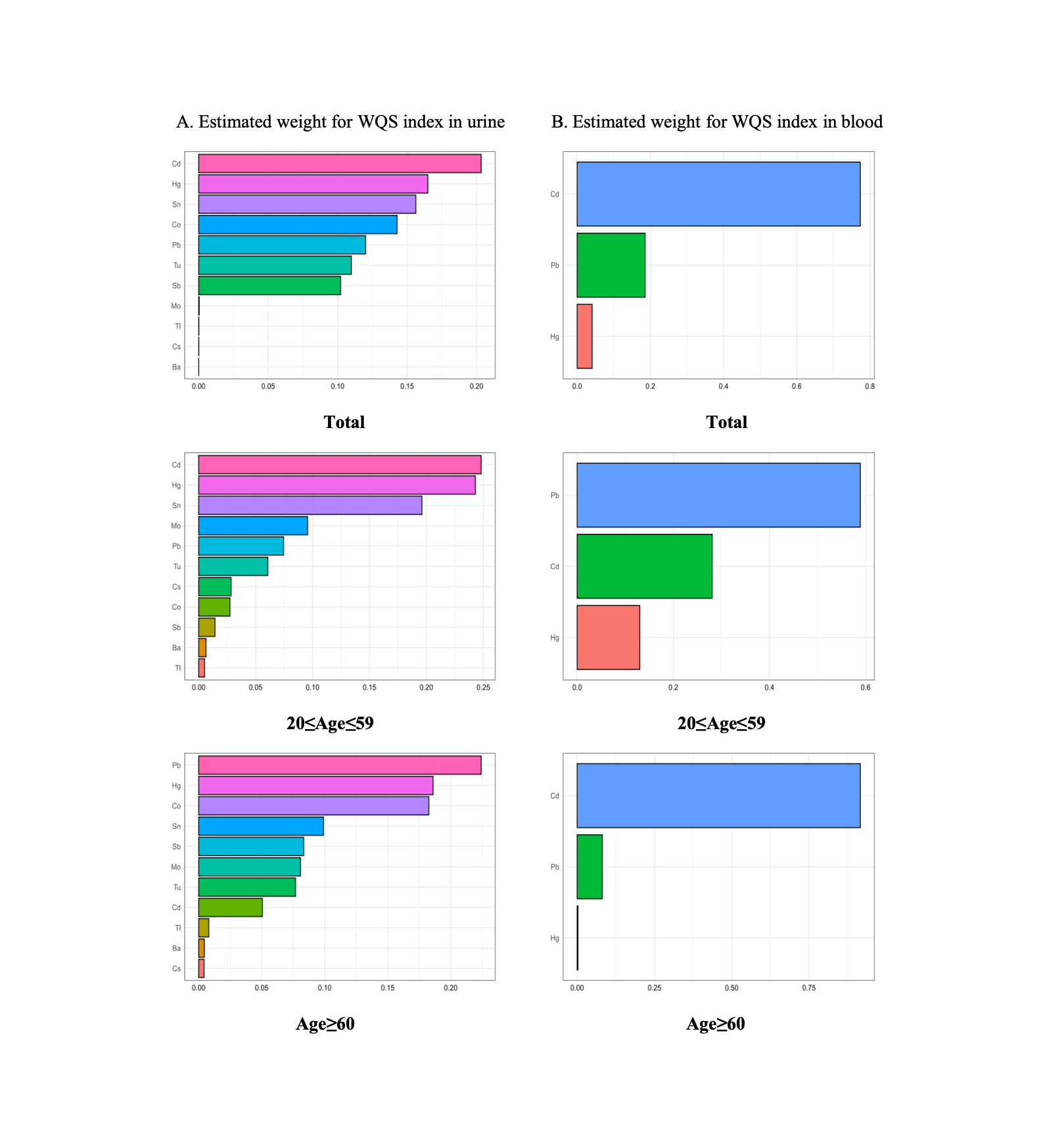


Figure S2. Estimated weights of urinary (A) and blood (B) metals for visual disability in WQS regression models adjusted for gender, age, race/ethnicity, education levels, poverty income ratio, marital status, body mass index, serum cotinine, and NHANES cycles.


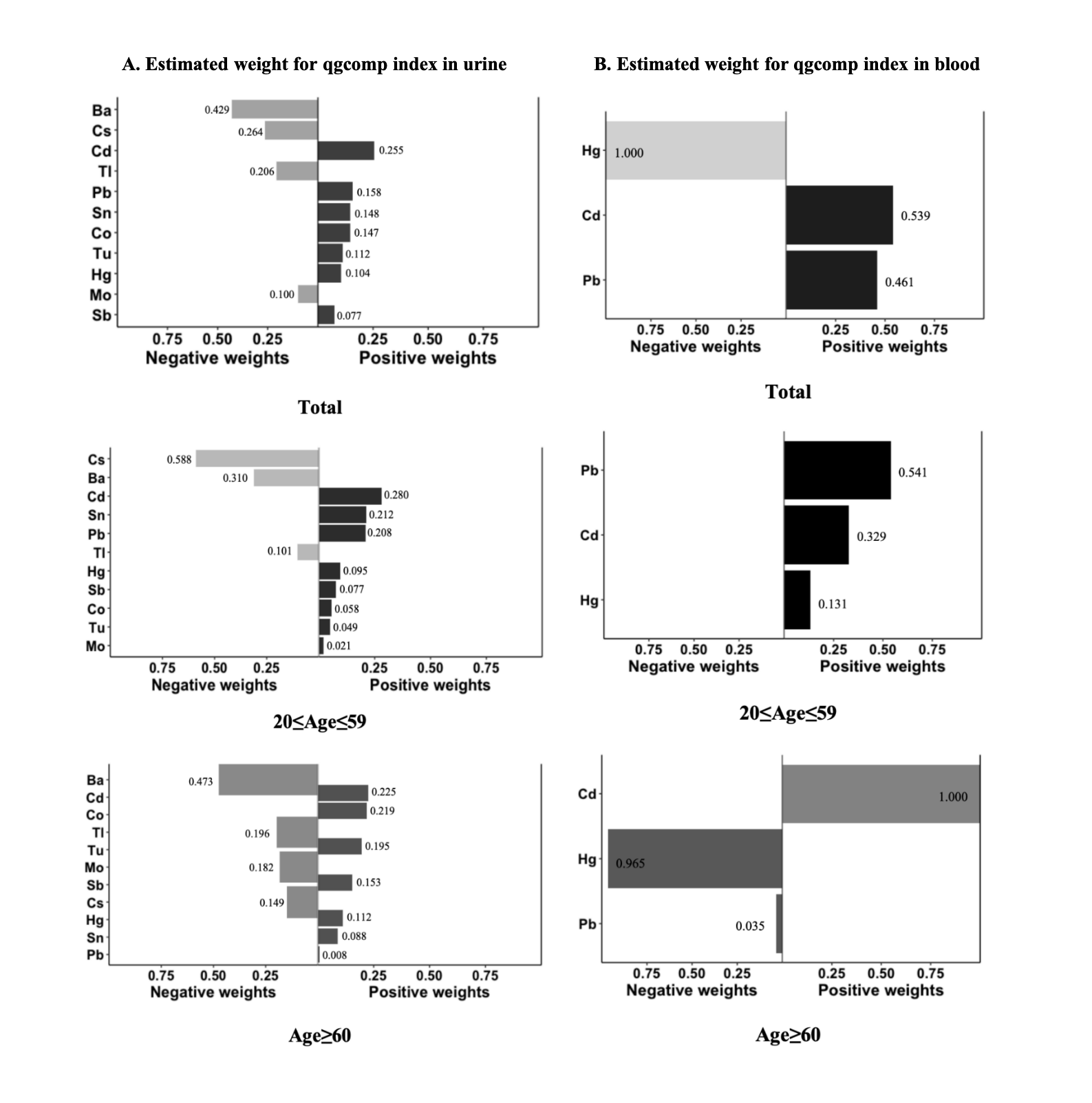


Figure S3. Estimated weights of each urinary (A) and blood (B) metal for visual disability in qgcomp regression models. Models were adjusted for gender, age, race/ethnicity, education levels, poverty income ratio, marital status, body mass index, serum cotinine, and NHANES cycles.


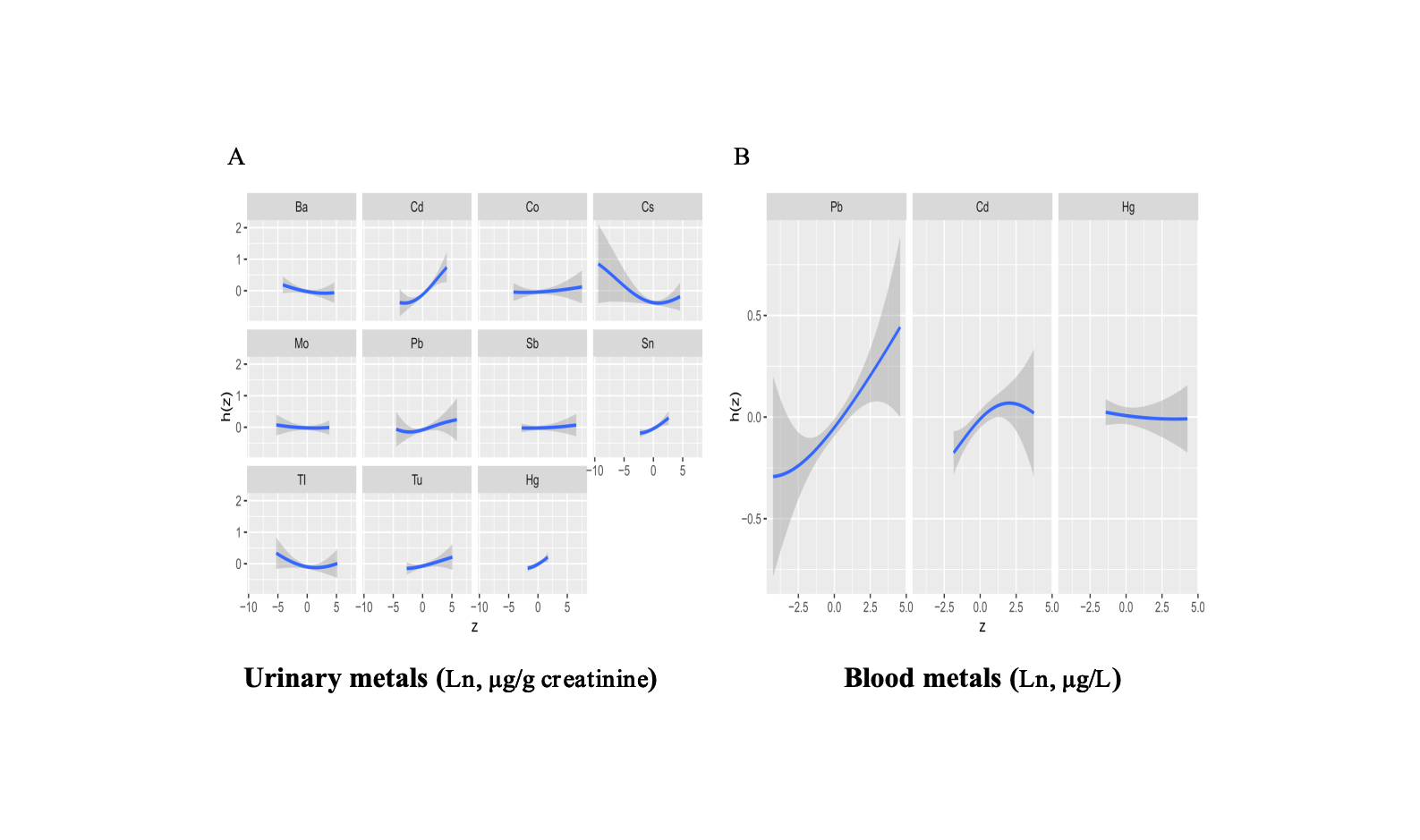
Figure S4. Univariate exposure-response plots urinary (A) and blood (B) metals for visual disability by BKMR models adjusted for gender, age, race/ethnicity, education levels, poverty income ratio, marital status, body mass index, serum cotinine, and NHANES cycles.

A B


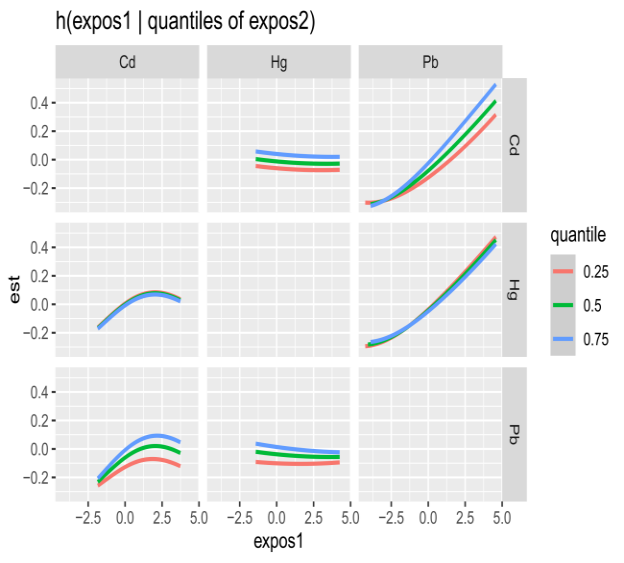

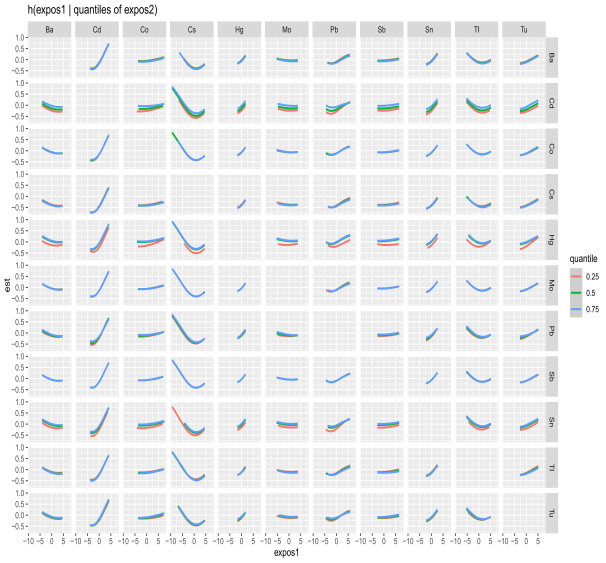
**Urinary metals (Ln, μg/g creatinine) Blood metals (Ln, μg/L)**

Figure S5. Binary exposure-response plots of urinary (A) and blood (B) metals for visual disability by BKMR models adjusted for gender, age, race/ethnicity, education levels, poverty income ratio, marital status, body mass index, serum cotinine, and NHANES cycles.

Table S1.The distributions of metal concentrations in urine and blood in the NHANES 2013-2018

| Metals | Detection  rates (%) | Total  (N= 4,284) |  | Non-visual disability  (N=4,000) |  | Visual disability (N=284) |
| --- | --- | --- | --- | --- | --- | --- |
|  |  | Median (IQR) |  | Median (IQR) |  | Median (IQR) |
| **Urine (μg/g creatinine)** | | | | | | |
| Ba | 99.5 | 0.96 (0.47-1.96) |  | 0.96 (0.48-1.98) |  | 0.84 (0.39-1.75) |
| Cd | 93.7 | 0.20 (0.10-0.40) |  | 0.19 (0.09-0.39) |  | 0.31 (0.15-0.58) |
| Co | 99.9 | 0.39 (0.23-0.64) |  | 0.39 (0.23-0.64) |  | 0.40 (0.25-0.59) |
| Cs | 100.0 | 4.30 (2.59-6.51) |  | 4.31 (2.61-6.53) |  | 4.10 (2.35-6.20) |
| Mo | 100.0 | 35.10 (18.10-62.64) |  | 34.93(18.10-62.50) |  | 40.42 (18.20-64.49) |
| Pb | 99.5 | 0.33 (0.18-0.57) |  | 0.32 (0.17-0.56) |  | 0.43 (0.21-0.78) |
| Sb | 77.0 | 0.04 (0.02-0.08) |  | 0.04 (0.02-0.07) |  | 0.05 (0.03-0.08) |
| Sn | 91.9 | 0.44 (0.20-1.00) |  | 0.43 (0.20-0.97) |  | 0.70 (0.33-1.60) |
| Tl | 99.5 | 0.16 (0.09-0.25) |  | 0.16 (0.09-0.25) |  | 0.13 (0.08-0.22) |
| Tu | 82.7 | 0.06 (0.03-0.11) |  | 0.06 (0.02-0.11) |  | 0.06 (0.03-0.11) |
| Hg | 60.0 | 0.18 (0.09-0.43) |  | 0.18 (0.09-0.43) |  | 0.17 (0.09-0.37) |
| **Blood (μg/L)** | | | | | | |
| Pb | 99.9 | 9.80 (6.10-15.50) |  | 9.60 (6.00-15.30) |  | 12.40 (8.43-19.88) |
| Cd | 92.7 | 0.30 (0.19-0.56) |  | 0.30 (0.18-0.54) |  | 0.41 (0.23-0.71) |
| Hg | 85.9 | 0.74 (0.40-1.58) |  | 0.75 (0.40-1.60) |  | 0.65 (0.38-1.23) |

IQR=inter-quartile range.

Table S2. The posteriori inclusion probability of single blood and urinary metals in the NHANES 2013-2018

| Metals | Total | 20 ≤ Age ≤ 59 | Age ≥ 60 |
| --- | --- | --- | --- |
| **Urine (μg/g creatinine)** | | | |
| Ba | 0.30 | 0.25 | 0.38 |
| Cd | 1.00 | 1.00 | 0.26 |
| Co | 0.21 | 0.13 | 0.16 |
| Cs | 0.57 | 0.43 | 0.17 |
| Mo | 0.19 | 0.06 | 0.19 |
| Pb | 0.64 | 0.31 | 0.27 |
| Sb | 0.16 | 0.02 | 0.15 |
| Sn | 0.67 | 0.40 | 0.31 |
| Tl | 0.37 | 0.22 | 0.17 |
| Tu | 0.58 | 0.15 | 0.31 |
| Hg | 0.50 | 0.25 | 0.27 |
| **Blood(μg/L)** | | | |
| Pb | 1.00 | 0.87 | 0.17 |
| Cd | 0.83 | 0.57 | 0.10 |
| Hg | 0.08 | 0.12 | 0.29 |
